# A Nanoparticle-Poly(I:C) Combination Adjuvant Enhances the Breadth of the Immune Response to Inactivated Influenza Virus Vaccine in Pigs

**DOI:** 10.3390/vaccines8020229

**Published:** 2020-05-18

**Authors:** Sankar Renu, Ninoshkaly Feliciano-Ruiz, Fangjia Lu, Shristi Ghimire, Yi Han, Jennifer Schrock, Santosh Dhakal, Veerupaxagouda Patil, Steven Krakowka, Harm HogenEsch, Gourapura J. Renukaradhya

**Affiliations:** 1 Food Animal Health Research Program, Ohio Agricultural Research and Development Center, 1680 Madison Avenue, Wooster, OH 44691, USA; renu.2@osu.edu (S.R.); feliciano-ruiz.1@buckeyemail.osu.edu (N.F.-R.); shristig@gmail.com (S.G.); han.1201@buckeyemail.osu.edu (Y.H.); schrock.57@osu.edu (J.S.); santoshdhakal88@gmail.com (S.D.); patil.202@osu.edu (V.P.); 2 Department of Veterinary Preventive Medicine, College of Veterinary Medicine, The Ohio State University, Columbus, OH 43210, USA; 3 Department of Comparative Pathobiology, College of Veterinary Medicine, Purdue University, West Lafayette, IN 47907, USA; aradhyagjr@gmail.com (F.L.); hogenesc@purdue.edu (H.H.); 4 The Department of Veterinary Biosciences, College of Veterinary Medicine, The Ohio State University, Columbus, OH 43210, USA; krakowkasummit@yahoo.com

**Keywords:** Nano-11, swine influenza virus, T and B cell peptides, poly(I:C), intranasal vaccination, mucosal immunity, pigs

## Abstract

Intranasal vaccination elicits secretory IgA (SIgA) antibodies in the airways, which is required for cross-protection against influenza. To enhance the breadth of immunity induced by a killed swine influenza virus antigen (KAg) or conserved T cell and B cell peptides, we adsorbed the antigens together with the TLR3 agonist poly(I:C) electrostatically onto cationic alpha-D-glucan nanoparticles (Nano-11) resulting in Nano-11-KAg-poly(I:C) and Nano-11-peptides-poly(I:C) vaccines. In vitro, increased TNF-α and IL-1ß cytokine mRNA expression was observed in Nano-11-KAg-poly(I:C)-treated porcine monocyte-derived dendritic cells. Nano-11-KAg-poly(I:C), but not Nano-11-peptides-poly(I:C), delivered intranasally in pigs induced high levels of cross-reactive virus-specific SIgA antibodies secretion in the nasal passage and lungs compared to a multivalent commercial influenza virus vaccine administered intramuscularly. The commercial and Nano-11-KAg-poly(I:C) vaccinations increased the frequency of IFNγ secreting T cells. The poly(I:C) adjuvanted Nano-11-based vaccines increased various cytokine mRNA expressions in lymph nodes compared to the commercial vaccine. In addition, Nano-11-KAg-poly(I:C) vaccine elicited high levels of virus neutralizing antibodies in bronchoalveolar lavage fluid. Microscopic lung lesions and challenge virus load were partially reduced in poly(I:C) adjuvanted Nano-11 and commercial influenza vaccinates. In conclusion, compared to our earlier study with Nano-11-KAg vaccine, addition of poly(I:C) to the formulation improved cross-protective antibody and cytokine response.

## 1. Introduction

Virulent swine influenza A virus (SwIAV) infection causes acute febrile respiratory disease in pigs of all ages, and is a serious economic burden to the global pork industry [1,2]. Pigs are highly susceptible to influenza virus infection owing to the presence of receptors for both mammalian (swine/human) and avian origin viruses in respiratory epithelial cells [3]. H1N1, H1N2, and H3N2 strains are the commonly circulating influenza virus subtypes in pigs [4]. The triple reassortant 2009 pandemic H1N1 SwIAV spillover to humans is evidence that pigs can act as a mixing vessel for mammalian and avian influenza viruses [2,3]. Continuous antigenic drift and shift in influenza viruses complicate disease control strategies [4]. It is accepted that developing SwIAV-specific cross-reactive immune response in pigs through vaccination is the most convenient and effective method to mitigate disease outbreaks, which will help the swine industry and reduce the public health risk.

The commercial inactivated SwIAV vaccine is a multivalent virus formulation for intramuscular (IM) injection. It induces specific IgG antibody responses and provides variable degrees of protection against field virus outbreaks [5]. However, the commercial vaccine is a poor inducer of secretory IgA (SIgA) antibodies in the airways where the virus actually enters the body and replicates. Virus-specific cell-mediated immune responses that contribute to clearing the mutated and reassorted SwIAVs are poor, presumably because antigens in the commercial vaccine do not enter the endogenous pathway of antigen presenting cells (APCs) [5,6]. Intranasally (IN)-delivered vaccines mimic the natural infection and effectively control influenza [7,8,9]. These vaccines primarily target nasal-associated lymphoid tissues (NALT) which have abundant APCs, T and B cells. Activated T and B cells in the NALT reach the effector site and elicit cross-reactive SIgA antibody and T cell responses [8,10]. However, IN-delivered inactivated or split virus antigens are poorly immunogenic, and they need a suitable adjuvant and/or vaccine delivery system to trigger the specific immune response [10].

Recently, we developed and characterized sweet corn-derived cationic alpha-D-glucan nanoparticles (Nano-11) and established its adjuvant potential in both mice and pigs [11,12,13]. Nano-11 is effectively phagocytized by dendritic cells, increases the expression of co-stimulatory molecules CD80 and CD86, and induces the secretion of IL-1β [12]. Protein antigen adsorbed to Nano-11 induced a comparable antibody response to standard aluminum hydroxide adjuvant upon intramuscular injection in mice [11]. In pigs, killed SwIAV antigen (KAg) adsorbed on Nano-11 (Nano-11-KAg) delivered IN induced cross-reactive SIgA response in the nasal passage but it did not induce IgG antibodies in the serum and lungs or cross-reactive T cell response [13]. SwIAV-KAg encapsulated in PLGA and polyanhydride nanoparticles delivered IN induced robust T cell response, but the antibody response was poor in pigs [14,15]. In another study, highly conserved influenza virus peptides encapsulated in PLGA or liposome nanoparticles delivered IN in pigs elicited epitope-specific T cell responses, but not antibody responses [16,17].

Poly(I:C) is a synthetic double stranded RNA molecule and a toll-like receptor (TLR)-3 ligand. Administration of poly(I:C) with vaccine antigens to mice activated innate type-I interferon (IFN-I) response and enhanced the antibody response [18,19]. Poly(I:C) adjuvanted SwIAV-KAg delivered IN in pigs induced a mucosal antibody response [20]. Here, we assessed the effect of a combination adjuvant composed of Nano-11 and poly(I:C) on the mucosal and systemic immune response, and protection induced by IN-delivered SwIAV-KAg (Nano-11-KAg-poly(I:C)), and a pool of viral peptides (Nano-11-peptides-poly(I:C)) in nursery pigs. The effect of these IN vaccines was compared with a multivalent commercial influenza vaccine.

## 2. Materials and Methods

### 2.1. Preparation of Influenza Viruses, Conserved Peptides, Adjuvant Poly(I:C) and Commercial Swine Flu Vaccine

Using Madin-Darby Canine Kidney epithelial (MDCK) cells, the field isolates of influenza viruses A/Swine/OH/FAH10-1/10 (H1N2-OH10), A/Swine/OH/24366/2007 (H1N1-OH7), and A/Turkey/OH/313053/2004 (H3N2-OH4) were propagated. Briefly, MDCK cells-grown virus cell-free supernatant was clarified, sucrose density gradient ultracentrifuged, and in phosphate-buffered saline (PBS), the virus pellet was suspended. Before virus inactivation using binary ethyleneimine, the virus titer was checked in MDCK cells [14]. Using a micro-BCA protein assay kit (Thermo Scientific, Waltham, MA, USA) antigen concentration was examined after inactivation and confirmed its non-replication on MDCK cells as described previously [14].

The influenza virus-specific conserved T cell and B cell peptides (Table 1) were custom synthesized by Ohio Peptide (Powell, OH, USA) and dissolved in recommended acidic and alkaline buffer and the aliquots (1 mg/mL) were stored at −80 °C until use as described previously [16]. Poly(I:C) HMW powder was acquired from Invivogen (Carlsbad, CA, USA), dissolved as per the company’s instructions, and aliquots were stored frozen. The FluSure XP^®^ commercial inactivated swine influenza virus vaccine was purchased from Zoetis (Kalamazoo, MI, USA); it is a multivalent commercial inactivated virus vaccine containing H1N1, H1N2 and H3N2 SwIAVs and an adjuvant.

### 2.2. Formulation of Nano-11-KAg-poly(I:C) and Nano-11-Peptides-Poly(I:C)

The Nano-11-based vaccines formulation was prepared as reported previously [12], with few modifications. For Nano-11-KAg-poly(I:C) preparation, 16 mg of Nano-11 powder was dissolved in 8 mL (2 mg/mL) of 3-(N-morpholino) propanesulfonic acid (MOPS) pH 7.4 buffer under magnetic stirring. The inactivated H1N2-OH10 SwIAV-KAg 2 mg in 1 mL MOPS buffer followed by 1.8 mg poly(I:C) (1 mg/mL) in endotoxin free water were added dropwise using an insulin syringe. Similarly, the Nano-11-peptides-poly(I:C) vaccine was prepared by using 40 mg Nano-11, 5 mg pooled peptides (0.5 mg each of 10 peptides) and 2.1 mg poly(I:C). After one hour of magnetic stirring, the Nano-11-KAg-poly(I:C) and Nano-11-peptides-poly(I:C) vaccines were obtained in the pellet after centrifugation at 10,976x *g* for 30 min, suspended in MOPS buffer and used for vaccination. The effect of adsorption on the particle size and zeta potential of the particles was determined in a Zeta-sizer coupled with an MPT-2 titrator (Malvern). The supernatant in the formulations was checked for the unbound KAg or peptides by using a micro-BCA protein assay kit, and poly(I:C) by measuring absorbance in the NanoDrop™ 2000c Spectrophotometer (Thermo Fisher Scientific, Waltham, MA, USA) as reported previously [12,14].

### 2.3. In Vitro Generation and Treatment of Porcine Monocyte-Derived Dendritic Cells (MoDCs)

The MoDCs were generated from peripheral blood mononuclear cells (PBMCs) as described previously [21] with some modifications. In brief, PBMCs were isolated from three conventional sows’ blood and plated at 10 million cells/well in RPMI containing 10% FBS in 12-well cell culture plates overnight at 37 °C in a 5% CO_2_ incubator. The floating cells were removed and attached cells were treated with stimulation medium containing cytokines GM-CSF (25 ng/mL) and IL-4 (10 ng/mL) (Kingfisher Biotech, Inc., Saint Paul, MN, USA). On the 3rd day, half the culture media was replaced with fresh cytokines containing stimulation medium. On day 6, medium was removed, all the cells were washed and treated with 1 mL of either medium (control), medium containing Nano-11 (80 μg/mL), Nano-11 (80 μg/mL) adsorbed with KAg (10 μg/mL), Nano-11 (80 μg/mL) adsorbed with poly(I:C) (10 μg/mL), and Nano-11 (80 μg/mL) adsorbed with both KAg (10 μg/ml) and poly(I:C) (10 μg/mL) [Nano-11-KAg; Nano-11-poly(I:C); and Nano-11-KAg-poly(I:C)] for 24 h at 37 °C. Total RNA was extracted from the treated cells and used for mRNA expression analyses by quantitative reverse transcription PCR as described below.

### 2.4. Vaccination and Virus Challenge Trial in Pigs

The vaccine trial in pigs was performed as reported previously [14]. Briefly, SwIAV and its antibody free caesarian-delivered colostrum-deprived piglets were raised in the Ohio Agricultural Research and Development Center biosafety level-2 facility. At the age of 5 weeks, male and female piglets (*n* = 23) were randomly distributed into five experimental groups as follows, (i) mock control (*n* = 4); (ii) soluble poly(I:C) (300 μg to each piglets) (*n* = 4); (iii) Nano-11-KAg-poly(I:C) (10^7^ TCID_50_ equivalent of KAg and 300 μg poly(I:C) to each piglets) (*n* = 5); (iv) Nano-11-peptides-poly(I:C) (50 μg each of 10 peptides and 300 μg poly(I:C) to each piglets) (*n* = 5); and (v) Commercial FluSure XP^®^ vaccine (*n* = 5). Experimental pigs were vaccinated IN through both the nostrils by using a spray mist delivery device (Prima Tech USA, NC) as reported previously [14]. The commercial vaccine was delivered IM as per the manufacturer’s instructions. After three weeks, pigs received booster dose like the first dose. Two weeks later, except the mock group, other experimental pigs were challenged with a virulent SwIAV SW/OH/24366/2007 (H1N1-OH7) 6 × 10^6^ TCID_50_ by both IN and intratracheal routes (50% virus delivered by each route). The virus challenged (Ch) pigs were monitored daily for clinical flu signs (fever, labored breathing, sneezing and reduced feed intake) and euthanized at day 6 post-challenge. During necropsy nasal swab, blood samples for serum and isolation of PBMCs, lung samples for preparing lung lysate and histopathology, bronchoalveolar lavage (BAL) fluid and tracheobronchial lymph nodes (TBLN) in RNA later were collected, processed and stored as reported previously [14].

### 2.5. Enzyme-Linked Immunosorbent Assay (ELISA) Assay

Detection of specific antibodies in various pig samples were carried out as reported previously [22]. Briefly, H1N2-OH10, H1N1-OH7, or H3N2-OH4 IAV KAg pre-titrated amounts (10 μg/mL) were coated in 96-well plates (Greiner bio-one, Monroe, NC, USA) and blocked with 5% skim milk containing 0.05% Tween-20 for 2 h at room temperature (RT) after overnight incubation at 4 °C. After three times washed with PBS Tween-20 (0.05%) (PBST) buffer, serial two-fold diluted nasal swab; lung lysate and BAL fluid for IgA; and serum, lung lysate and BAL fluid for IgG antibodies detection were added to marked duplicate wells and incubated overnight at 4 °C. Plates were washed with PBST and horseradish peroxidase conjugated goat anti-pig IgA (Bethyl Laboratories, Montgomery, TX, USA) or goat anti-pig IgG (KPL, Gaithersburg, MD, USA) was added. After 2 h incubation at RT, plates were washed with PBST and 1:1 mixture of peroxidase substrate solution B and TMB peroxidase substrate (KPL, Gaithersburg, MD, USA) was added and the reaction was stopped after 10–15 of incubation by adding 1 M phosphoric acid solution. Using the ELISA Spectramax microplate reader (Molecular devices, San Jose, CA, USA), the optical density (OD) values were measured at 450 nm. The corrected OD value was obtained after subtraction of the blank value.

### 2.6. Flow Cytometry Analyses

The isolated PBMCs were stimulated with vaccine virus (H1N2-OH10) at 0.1 multiplicity of infection (MOI) for 48 h and immunostained for different lymphocyte subsets as described previously [14]. In brief, cells were blocked with pig serum and surface stained with pig lymphocyte-specific monoclonal antibodies. For intracellular IFNγ staining, GolgiPlug™ (BD Biosciences, San Jose, CA, USA) and Brefeldin A (Sigma-Aldrich, St. Louis, MO, USA) were added during the last 6 h of stimulation. The immunostained cells were fixed with 1% paraformaldehyde and permeabilized with cell permeabilization buffer (85.9% deionized water, 11% PBS without Ca^2+^ or Mg^2+^, 3% formaldehyde solution, and 0.1% saponin), washed and acquired 50,000 events in BD Aria II flow cytometer (BD Biosciences, San Jose, CA, USA). Using FlowJo software (Tree Star, Palo Alto, CA, USA), the data were analyzed. Fluorochrome-labeled antibodies used in flow cytometry were anti-porcine CD3 (PerCP), CD4α (APC- Cy7) and CD8α (FITC) procured from SouthernBiotech (Birmingham, AL, USA), and anti-CD8β (PE-Cy7), δ chain (APC) and IFNγ (PE) monoclonal antibodies from BD Biosciences (San Jose, CA, USA).

### 2.7. Quantitative Reverse Transcription PCR (qRT-PCR) Analyses

Total RNA was extracted from the treated MoDCs and TBLN using TRIzol reagent (Invitrogen, Carlsbad, CA, USA). The cDNA was converted from 1 or 2 μg of total RNA and the target genes TNF-α, IL-1ß, GATA3, IL-2, IL-6, IL-10 and IL-13, and housekeeping gene β-actin (Table 2) expression were attained by qRT-PCR (Applied Biosystems, Foster City, CA, USA) using the SYBR Green Supermix kit (Bio-Rad Laboratories, Hercules, CA, USA) [23]. The fold changes were calculated as described previously [24].

### 2.8. Virus Neutralization Test (VNT) Titer and Infectious Virus Titration

The assays were performed as reported previously [14,17]. Heat inactivated BAL fluid and serum samples were used for VNT. A serial two-fold diluted BAL fluid and serum samples were incubated with 100 TCID_50_ of the H1N1-OH7 virus. The suspension was transferred into confluent MDCK cells in a 96-well plate and incubated. For virus titration, ten-fold serial diluted nasal swab, BAL fluid and lung lysate samples were added into MDCK cells and incubated for 48 h. The cells were fixed, immunostained with influenza virus nucleoprotein-specific antibody (CalBioreagents, Foster City, CA, USA) and AlexaFluor 488 conjugated goat anti-mouse IgG (H+L) antibody (Life Technologies, Carlsbad, CA, USA). The VNT titer and infectious virus titer-specific immunofluorescence were examined in a fluorescent microscope (IX51, Olympus, Tokyo, Japan) and the titers were calculated.

### 2.9. Histopathology of Lungs

The procedure was performed as described previously [14]. Using 10% natural buffer formalin, the lung lobes were infused, processed, and 5 μm sections of apical, cardiac and diaphragmatic lobes were stained with hematoxylin and eosin (H&E). Stained slides were examined microscopically for interstitial pneumonia, bronchial exudates, and peribronchial and perivascular accumulation of mononuclear inflammatory cells-based lesions by a board certificated veterinary pathologist (SK). The lesions were scored as follows: 0—no changes; 0.5—observed changes but too mild; 1—minimal changes; 2—moderate changes; 3—marked changes. The final lung lesions score of each pig was determined by taking the average of three lung lobes score. Representative lung images of each experimental group were taken using a phase contrast microscope.

### 2.10. Statistical Analyses 

Statistical analyses of ELISA data were carried out using two-way ANOVA followed by a Bonferroni test using the GraphPad Prism 5 (GraphPad Software, Inc., San Diego, CA, USA). The remaining data were analyzed by adapting one-way analysis of variance followed by Tukey’s post hoc comparison test. A *p* < 0.05 was considered statistically significant. Data were presented as the mean ± SEM of four to five pigs.

## 3. Results

### 3.1. Preparation of Poly(I:C) Adjuvanted Nano-11-Based Influenza Vaccines

We evaluated different ratios of Nano-11 and KAg as a model antigen (1:1, 2:1, 4:1 and 8:1) to prepare the optimal vaccine formulation based on nanoparticle size, surface charge and antigen adsorption efficiency. We observed that 8:1 ratio of Nano-11 and KAg provided particles of 214 nm size, net positive surface charge of 20.4 mV, and 93% KAg adsorption efficiency. The 2:1 ratio yielded 487 nm size particles, +19.2 mV charge and 84.2% KAg adsorption efficiency. Thus, based on our initial formulation optimization study results we used the 8:1 ratio of Nano-11 and KAg or pooled ten peptides to prepare the vaccine formulation to use in an in vitro, and in vivo vaccine trial in pigs. Poly(I:C) was adsorbed 100% on Nano-11. The poly(I:C) adjuvanted vaccines, Nano-11-KAg-poly(I:C) and Nano-11-peptides-poly(I:C), had approximately 93% and 72% antigen adsorption efficiency, respectively.

### 3.2. Nano-11-KAg-poly(I:C) Treatment Increased the Innate and Th1 Cytokine mRNA Expression in MoDCs

**Figure 1:**
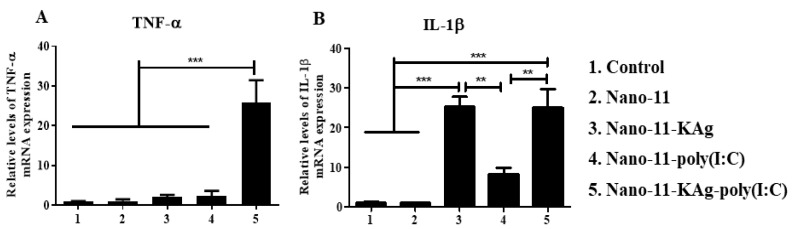
Porcine monocyte-derived dendritic cells (MoDCs) treated with Nano-11-KAg-poly(I:C) increased the cytokine mRNA expression. Porcine PBMCs-derived MoDCs were treated with medium (control), Nano-11, Nano-11-KAg, Nano-11-poly(I:C), and Nano-11-KAg-poly(I:C) for 24 h and different cytokine mRNA expressions were analyzed by qRT-PCR. The fold-change in mRNA expression levels of (**A**) TNF-α; and (**B**) IL-1ß was determined. Each bar is the mean ± SEM of three pigs and the data were analyzed by one-way ANOVA followed by Tukey’s post hoc comparison test. Asterisk refers to statistical difference between the indicated treatment groups (** *p* < 0.01 and *** *p* < 0.001).

Initially to evaluate whether binding of poly(I:C) on Nano-11 enhances the activation of APCs, and poly(I:C) does not interfere with co-adsorbed KAg binding, an in vitro experiment was performed on MoDCs. The MoDCs treated with Nano-11-KAg-poly(I:C) had a significantly augmented (*p* < 0.001) TNF-α mRNA expression compared to all the controls such as Nano-11, Nano-11-KAg and Nano-11-poly(I:C) (Figure 1A). The IL-1ß mRNA expression was significantly enhanced (*p* < 0.001 and *p* < 0.01) in both Nano-11-KAg and Nano-11-KAg-poly(I:C) treated cells compared to the other three controls, including the Nano-11-poly(I:C) (Figure 1B).

### 3.3. Nano-11-KAg-poly(I:C) Nanovaccine Augmented Cross-Reactive SIgA Antibody Response

**Figure 2:**
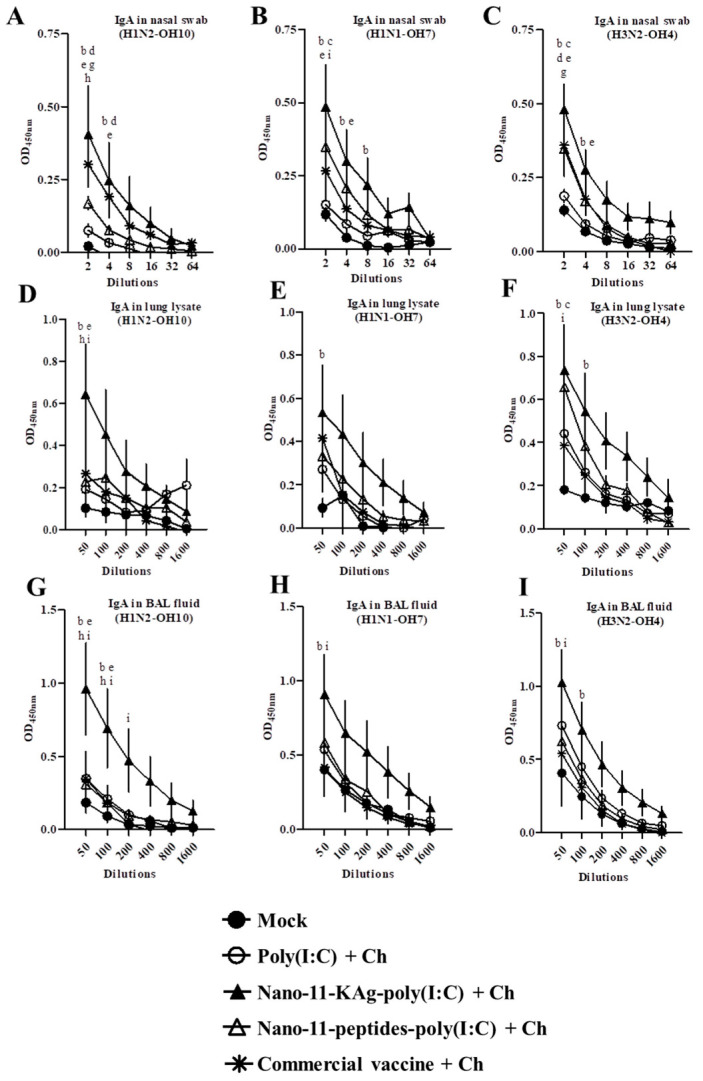
Poly(I:C) adjuvanted Nano-11-KAg vaccine induced increased cross-reactive SIgA antibody response. Pigs were vaccinated twice with poly(I:C) adjuvanted Nano-11-based vaccines (containing H1N2-OH10 virus or 10 peptides) or commercial vaccine (containing H1N1, H1N2 and H3N2 viruses) and challenged (H1N1-OH7 virus) at 35 days post prime vaccination. Samples collected at day six post-challenge were used for SIgA antibody analysis. Secretory IgA antibody response in nasal swab, lung lysate, and BAL fluid samples against (**A**,**D**,**G**) H1N2-OH10; (**B**,**E**,**H**) H1N1-OH7; and (**C**,**F**,**I**) H3N2-OH4 viruses were analyzed by ELISA. Data represent the mean value of four to five pigs ± SEM at all indicated dilutions. Statistical analysis was carried out using two-way ANOVA followed by a Bonferroni test. Each letter indicates the significant difference between the groups at the indicated dilution. b, c, and d indicate the difference between mock group compared to Nano-11-KAg-poly(I:C) + Ch, Nano-11-peptide-poly(I:C) + Ch, and Commercial vaccine +Ch, respectively. e and g indicate the difference between poly(I:C) + Ch compared to Nano-11-KAg-poly(I:C) + Ch, and Commercial vaccine +Ch, respectively. h and i indicate the difference between Nano-11-KAg-poly(I:C) + Ch compared to Nano-11-peptide-poly(I:C) + Ch and Commercial vaccine +Ch, respectively. A *p* < 0.05 was considered statistically significant. Ch—Challenge.

Homologous (H1N2-OH10), heterologous (H1N1-OH7), and heterosubtypic (H3N2-OH4) influenza viruses antigen-specific cross-reactive SIgA antibody levels were increased in Nano-11-KAg-poly(I:C) vaccinated pigs at the tested serial dilutions (not in last two dilutions) of nasal swab, lung lysate, and BAL fluid samples compared to other vaccinates (Figure 2A–I). In Nano-11-KAg-poly(I:C), Nano-11-peptides-poly(I:C), and commercial vaccines-delivered pigs significantly increased (*p* < 0.05) H1N2-OH10, H1N1-OH7, and H3N2-OH4 virus-specific SIgA antibody levels in nasal swab samples at 1:2 dilution were observed (Figure 2A–C). The cross-reactive SIgA level in nasal swab was higher in the group vaccinated with Nano-11-KAg-poly(I:C) than in the other vaccine groups (Figure 2A–C).

The H1N2-OH10 and H1N1-OH7, but not H3N2-OH4 virus-specific SIgA antibody levels in lung lysate samples (represents response in lung parenchyma) were significantly increased (*p* < 0.05) in the Nano-11-KAg-poly(I:C) group compared to the commercial vaccine group (Figure 2D–F). Similarly, Nano-11-KAg-poly(I:C) vaccinated animals compared with the soluble poly(I:C) with challenge (mock-challenge) and commercial vaccine groups had significantly increased H1N2-OH10, H1N1-OH7, and H3N2-OH4 virus-specific SIgA in BAL fluid (*p* < 0.05) (Figure 2G–I).

### 3.4. Nano-11-KAg-poly(I:C) Nanovaccine Increased the IgG Antibody Response in Lungs But Not in Serum

**Figure 3:**
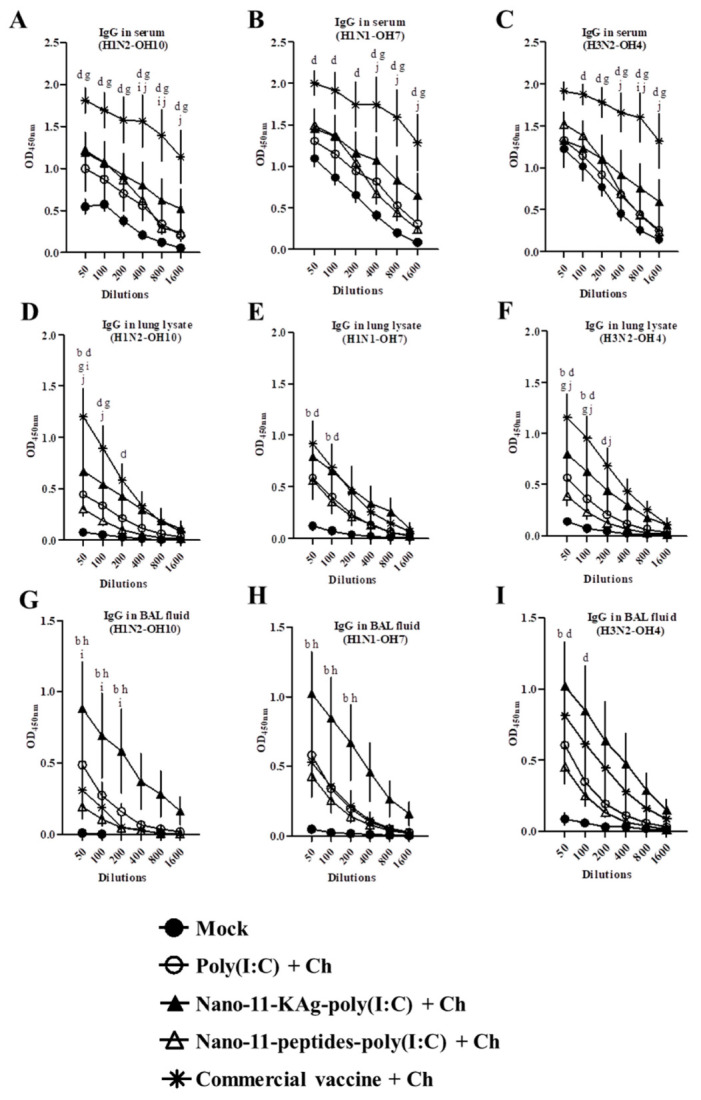
Commercial influenza vaccine augmented systemic IgG response and Nano-11-KAg-poly(I:C) vaccine in lower respiratory tract. Pigs were vaccinated twice with poly(I:C) adjuvanted Nano-11-based vaccines (containing H1N2-OH10 virus or 10 peptides) or commercial vaccine (containing H1N1, H1N2 and H3N2 viruses) and challenged (H1N1-OH7 virus) at 35 days post prime vaccination. Samples collected at day six post-challenge were used for IgG antibody analysis. The IgG antibody response in serum, lung lysate, and BAL fluid samples against (**A**,**D**,**G**) H1N2-OH10; (**B**,**E**,**H**) H1N1-OH7; and (**C**,**F**,**I**) H3N2-OH4 viruses were analyzed by ELISA. Data represent the mean value of four to five pigs ± SEM at all indicated dilutions. Statistical analysis was carried out using two-way ANOVA followed by a Bonferroni test. Each letter indicates the significant difference between the groups at the indicated dilution. b and d indicate the difference between mock group compared to Nano-11-KAg-poly(I:C) + Ch, and Commercial vaccine +Ch, respectively. g indicate the difference between poly(I:C) + Ch compared to Commercial vaccine +Ch. h and i indicate the difference between Nano-11-KAg-poly(I:C) + Ch compared to Nano-11-peptide-poly(I:C) + Ch and Commercial vaccine +Ch, respectively. j indicates difference between Nano-11-peptide-poly(I:C) + Ch) compared to Commercial vaccine +Ch. A *p* < 0.05 was considered statistically significant. Ch—Challenge.

Serum IgG antibody response against H1N2-OH10, H1N1-OH7, and H3N2-OH4 viruses were significantly increased (*p* < 0.05) in commercial vaccine immunized pigs compared to the other vaccine groups (Figure 3A–C). However, both the Nano-11-KAg-poly(I:C) and commercial vaccine induced significantly increased (*p* < 0.05) IgG antibody levels in the lung parenchyma against the H1N2-OH10, H1N1-OH7, and H3N2-OH4 viruses compared to mock pigs (Figure 3D–F). The IgG antibody levels in BAL fluid against H1N2-OH10, H1N1-OH7, and H3N2-OH4 viruses were increased at all the tested dilutions in Nano-11-KAg-poly(I:C) immunized pigs compared to the other groups including the commercial vaccine (Figure 3G–I). Notably, the H1N2-OH10 specific IgG antibody response in BAL fluid was significantly increased (*p* < 0.05) in the Nano-11-KAg-poly(I:C) group compared with animals that received the commercial vaccine (Figure 3G).

### 3.5. IFNγ Secretion by Lymphocytes of Poly(I:C) Adjuvanted Nano-11-Based Influenza Nanovaccinates

**Figure 4:**
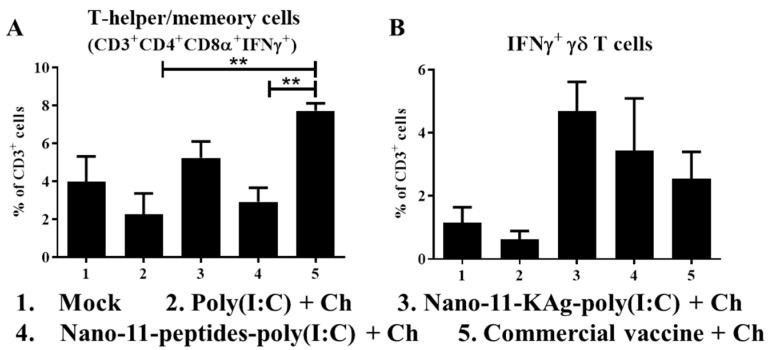
Recall IFN-γ secreting lymphocyte response in Nano-11 and commercial influenza vaccinated and virus challenged pigs. PBMCs isolated at post-challenge day six were stimulated with vaccine H1N2-OH10 SwIAV, immunostained and analyzed for the frequency of IFN-γ secreting (**A**) T-helper/memory cells (CD3^+^CD4^+^CD8α^+^); and (**B**) γδ T cells by flow cytometry. Data represent the mean value of four to five pigs ± SEM. Statistical analysis was carried out using one-way analysis of variance followed by Tukey’s post hoc comparison. Asterisk refers to statistical difference between the two indicated groups (** *p* < 0.01). Ch—Challenge.

The frequency of IFNγ secreting T-helper/memory (CD3^+^CD4^+^CD8α^+^) cells was significantly increased (*p* < 0.01) in animals vaccinated with the commercial vaccine (Figure 4A). On the other hand, a higher frequency of IFNγ secreting γδ T cells was noticed in Nano-11-KAg-poly(I:C) vaccinates, although this did not reach statistical significance (Figure 4B). However, Nano-11-KAg-poly(I:C) inoculation did not increase IFNγ secreting CD3^+^CD4^-^CD8αβ+ and CD3^+^CD4^+^CD8α^-^ T cell population (data not shown).

### 3.6. Poly(I:C) Adjuvanted Nano-11-Based Influenza Vaccines Increased Th1 and Th2 Cytokines mRNA Expression in TBLN

**Figure 5:**
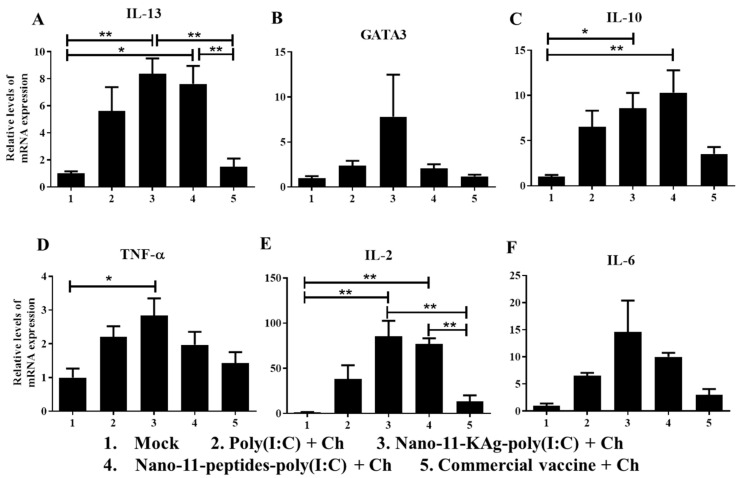
Cytokine and transcription factor mRNA expression in the tracheobronchial lymph nodes of pigs vaccinated with Nano-11 or commercial influenza vaccine and virus challenged. The mRNA expression levels of (**A**) IL-13; (**B**) GATA3; (**C**) IL-10; (**D**) TNF-α; (**E**) IL-2; and (**F**) IL-6 was determined by qRT-PCR. Data represent the mean value of four to five pigs ± SEM. Statistical analysis was carried out using one-way analysis of variance followed by Tukey’s post hoc comparison. Asterisk refers to statistical difference between the two indicated groups (* *p* < 0.05, and ** *p* < 0.01). Ch—Challenge.

We determined the mRNA expression of the cytokines IL-2, IL-6, TNF-α, IL-10 and IL-13 as well as the transcription factor GATA3 in the TBLN. IL-13 and GATA3 are associated with Th2 responses and IL-2 and TNF-α with Th1 responses, whereas IL-10 is an immunosuppressive cytokine and IL-6 a proinflammatory cytokine. Both Nano-11-KAg-poly(I:C) and Nano-11-peptides-poly(I:C) immunization significantly increased (*p* < 0.05 and *p* < 0.01) IL-13 mRNA compared to the commercial vaccine and mock groups (Figure 5A). Nano-11-KAg-poly(I:C) vaccine also had increased GATA3 mRNA expression compared to other groups (Figure 5B). The expression of IL-10 mRNA was significantly higher (*p* < 0.05 and *p* < 0.01) in both the Nano-11-based influenza vaccines group compared with the mock group (Figure 5C). TNF-α cytokine gene expression was increased in Nano-11-KAg-poly(I:C) vaccinates compared to other groups, and it was significantly increased (*p* < 0.05) compared with the mock group (Figure 5D). Notably, both Nano-11-KAg-poly(I:C) and Nano-11-peptides-poly(I:C) groups had significantly increased (*p* < 0.01) IL-2 mRNA expression compared to the mock and commercial vaccine administered animals (Figure 5E). IL-6 gene expression was increased in both Nano-11-containing influenza vaccine groups compared to other vaccinates (Figure 5F).

### 3.7. Nano-11-KAg-poly(I:C) Nanovaccinations Increased the VNT Titers in the Lung (But Not in Serum) with Comparable Virus Load in the Airways to That of Commercial Vaccine

**Figure 6:**
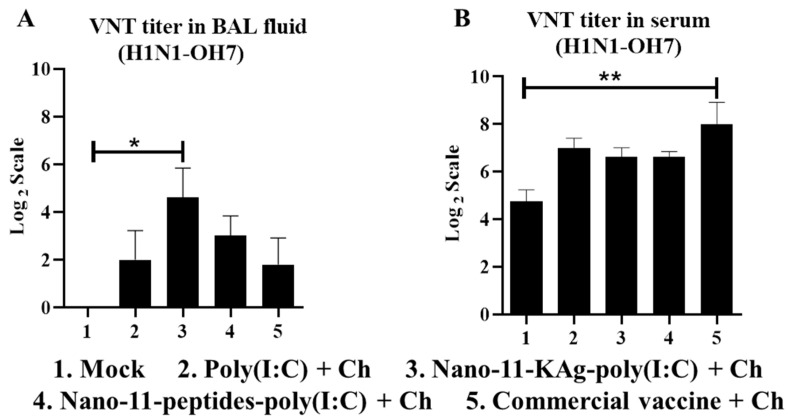
Nano-11-KAg-poly(I:C) and commercial influenza vaccines induced increased virus neutralization test titers in BAL fluid and serum of pigs, respectively. The H1N1-OH7 challenge virus-specific virus neutralization antibody titer in (**A**) BAL fluid; and (**B**) Serum samples collected at post-challenge day 6. Data represent the mean value of four to five pigs ± SEM. Statistical analysis was carried out using one-way analysis of variance followed by Tukey’s post hoc comparison. Asterisk refers to statistical difference between the two indicated groups (* *p* < 0.05, and ** *p* < 0.01). Ch—Challenge.

**Figure 7:**
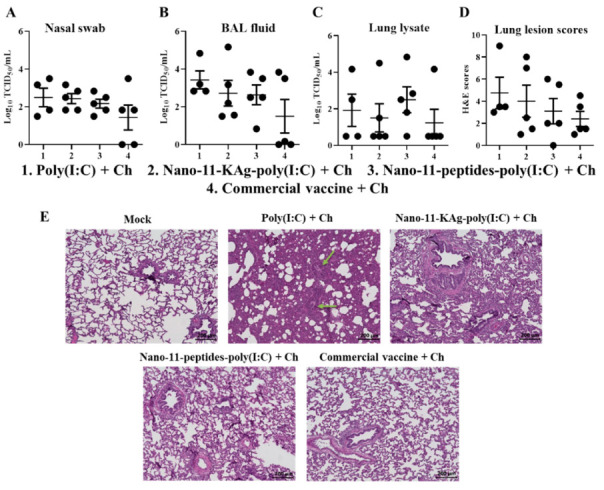
Nano-11 and commercial influenza vaccines reduced the challenge virus titer and virus induced lung pathology. Pigs were vaccinated twice with poly(I:C) adjuvanted Nano-11-based vaccines (containing H1N2-OH10 virus or 10 peptides) or commercial vaccine (containing H1N1, H1N2 and H3N2 viruses) and challenged (H1N1-OH7 virus) at 35 days post prime vaccination. Samples collected at day six post-challenge were used in the analyses. Challenge live SwIAV titer in (**A**) Nasal swab; (**B**) BAL fluid; and (**C**) Lung lysate. (**D**) Severity of lung lesions based on evaluation of hematoxylin and eosin-stained sections; and (**E**) Representative lung sections of experimental pigs in each group stained with hematoxylin and eosin were examined and microscopic images were taken at 10x magnification (green arrows show the sites of inflammation). Data represent the mean value of four to five pigs ± SEM. Ch—Challenge.

The Nano-11-KAg-poly(I:C) vaccine elicited enhanced SIgA and IgG antibodies levels in the lungs of pigs which was reflected in significantly higher (*p* < 0.05) VNT titer in the BAL fluid (Figure 6A). Corresponding to high specific IgG antibody response induced by the commercial vaccine in serum, a significantly higher (*p* < 0.01) VNT titer was observed (Figure 6B). The virulent H1N1-OH7 challenge virus load in the nasal swab, BAL fluid, and lung lysate samples of both Nano-11 and commercial influenza vaccines administered groups were partially reduced, with numerical (but not statistically significant) reduced load by the commercial vaccine compared to other groups (Figure 7A–C). We did not observe any influenza related visible clinical signs in any of the virus challenged experimental pigs, while the microscopic lung lesions such as interstitial pneumonia, bronchial exudate, peribronchial, and perivascular inflammation were partially reduced by both Nano-11-based and commercial influenza vaccines received pigs (Figure 7D,E).

## 4. Discussion

Polysaccharide structured Nano-11 is derived from the kernel of a genetic variant of sweet corn, *sugary-1* [12]. A simple chemical modification of the surface was used to synthesize the amphiphilic and cationic Nano-11 [12]. Mice injected IM with Nano-11 developed a moderate local inflammatory response, which resolved in about two weeks with no signs of systemic distribution or long-term deposition of particles at the injection site, suggesting its safety in animals [11]. We did not observe any clinical signs of adverse reactions after IM or IN administration of the Nano-11 formulated vaccines to pigs [13]. Nano-11-KAg delivered IN with no additional adjuvant induced cross-reactive SIgA response only in the nasal passage but not in BAL fluid, and failed to elicit systemic IgG and cross-reactive T cell responses [13]. Therefore, in this study, our goal was to improve the efficacy of Nano-11 influenza vaccines by incorporating the adjuvant poly(I:C) in the formulation, and also to evaluate the responses to conserved peptides delivered in Nano-11.

The positively charged Nano-11 readily adsorbs negatively charged molecules such as protein antigens and nucleic acids [12]. Increasing the ratio of Nano-11 to antigen leads to high antigen adsorption efficiency and smaller nanometer sized particles [13]. In this study, high Nano-11 to KAg ratio (8:1) was used compared to our earlier preparation (2:1 ratio), which resulted in approximately 10% higher KAg adsorption efficiency and reduced particle size from 487 to 214 nm compared with a 2:1 ratio [13]. Besides, further studies are needed using greater than 8:1 Nano-11 to KAg ratio to determine whether increasing the ratio alters the particle size and antigen adsorption efficiency, and importantly, the immunogenicity. The adsorption efficiency of peptides was lower than for KAg. The adsorption did not cause much change in the net positive surface charge on the Nano-11 vaccines.

The nasal passage is the primary gateway of influenza virus entry and secreted specific SIgA antibodies in the airways minimize the entry of virus to susceptible cells in the body [25,26]. Compared to our previous study in pigs using Nano-11-KAg [13], the Nano-11-KAg-poly(I:C) formulation induced enhanced cross-reactive SIgA secretion in the BAL fluid and IgG antibodies in serum and lungs. In addition, both the Nano-11-KAg-poly(I:C) and Nano-11-peptides-poly(I:C) formulations induced Th1 and Th2 cytokines mRNA expression in TBLN. Five conserved influenza virus B cell peptides incorporated in Nano-11-peptides-poly(I:C) vaccine failed to elicit a substantial antibody response either in the airways or in blood of pigs. Similarly, in our earlier study identical influenza virus peptides encapsulated in PLGA and liposome nanoparticles delivered IN failed to elicit antibody response [16,17].

As expected, the commercial IM-delivered vaccine induced a robust systemic serum IgG response and also IgG in the lung parenchyma, but it did not induce significant SIgA secretion in the airways [20,27]. An IN-delivered influenza vaccine induced high avidity SIgA antibodies and provided protection against a heterologous influenza challenge in mice, while the injectable vaccine elicited serum IgG but no SIgA [27]. The combination of nanoparticles and TLR ligands can elicit broad and strong humoral immunity through the expansion of T follicular helper cells in the germinal center [28]. Poly(I:C) co-administered with inactivated influenza virus IN induced cross-reactive SIgA and systemic IgG antibodies, while parenteral delivery failed to elicit SIgA secretion in mice [18,29,30]. In mice, influenza specific IgG acts as a backup for SIgA antibody-mediated influenza virus protection in the nasal passages, whereas IgG antibody is dominant in the lung [25]. Local airway SIgA and systemic IgG levels in chickens increased upon co-delivery of a TLR agonist with an inactivated avian influenza virus [31]. Moreover, co-administration of soluble poly(I:C) with SwIAV-KAg IN induced SIgA and serum IgG antibody response comparable to a commercial vaccine in pigs [20].

In this study, the group that received soluble poly(I:C) only and challenged with virus is our mock-challenge infection pig group, because poly(I:C) alone does not induce viral antigen-specific immunity to a challenge virus inoculated two weeks later. This is based on multiple previous vaccine trials that included challenge infections to naïve pigs [14,15,16,17,22]. The response in naïve animals was similar to the group that received poly(I:C) only.

The cell-mediated immune response is needed to rescue infected animals and to prevent complications associated with influenza [31]. Immunity to influenza virus in pigs is mainly mediated by T-helper/memory cells which possess cytolytic functions [14,32,33]. To clear influenza virus from the lungs of pigs, IFNγ secreted by cytotoxic T lymphocytes (CTLs), T-helper/memory, and γδ T cells play a major role [14]. Both the Nano-11-KAg-poly(I:C) and Nano-11-peptides-poly(I:C) vaccines enhanced the IFNγ secreting γδ T cell population, while the commercial vaccine increased the IFNγ secreting T-helper/memory cells frequency. In an earlier study, Nano-11-KAg without poly(I:C) induced both CTLs and T-helper/memory cells response specific to vaccine virus [13], while our current studies show that the addition of poly(I:C) to Nano-11-KAg triggered only γδ T cell responses along with Th1 and Th2 cytokines mRNA expression in TBLN. Influenza virus delivered IN with TLR3 agonist induced a similar homologous virus-specific T cell response in mice and pigs [9,29].

The induction of IL-2, IL-4, IL-10, TGFβ, and IL-21 cytokines promotes B cell proliferation and specific class switched plasma cells with long-lived memory B cells [26]. Addition of poly(I:C) in Nano-11-KAg augmented in vitro TNF-α and IL-1β mRNA expression in treated MoDCs, and in vivo in vaccinated pig lymphoid tissues (TBLN) observed enhanced IL-2, IL-13, IL-10 and TNF-α gene expression. While in our earlier study in Nano-11-KAg vaccinated pigs, any such cytokines gene expression was not detected in TBLN, though in vitro treated MoDCs secreted TNFα, IL-1β, IL-10, IL-6 and IL-12 cytokines [13]. Based on this study, it is clear that the poly(I:C) adsorbed on Nano-11-KAg or Nano-11-peptides enhanced Th1 and Th2 cytokines gene expression which likely augmented humoral and cell-mediated immune responses compared to earlier study with only Nano-11-KAg [13]. Similarly, in mice, poly(I:C) administered with influenza virus antigen IN augments IFNα, INFß, IFNγ, IL-4, IL-6, and IL-12 p40 mRNA expression [30]. In chickens, vaccination with avian influenza virus and poly(I:C) enhanced IL-6, IL-12, and IFNγ cytokine gene expression compared to non-adjuvanted vaccination [31]. We recently demonstrated that IN co-administration of chitosan nanoparticle loaded with influenza KAg or poly(I:C) in pigs induced enhanced Th1 and Th2 cytokines mRNA expression [9].

It is important to note that the multivalent inactivated influenza commercial vaccine used for comparative analysis in our study contains H1N1, H1N2, and H3N2 SwIAVs. The hemagglutinin (HA) gene sequence analysis data revealed that the H1N1-OH07 challenge virus has 95.2% and 77.9% HA genetic identity to the commercial vaccine H1N1 and H1N2 viruses, respectively. Whereas our Nano-11-KAg vaccine containing only the H1N2-OH10 virus with 77% HA genetic identity to the H1N1-OH07 challenge virus. This suggests that the commercial vaccine elicited immune responses mostly against the homologous virus, while Nano-11-KAg-poly(I:C) vaccine induced significant cross-reactive antibody and cytokine responses. Microscopic lung lesions and challenge virus load were partially reduced in both poly(I:C) adjuvanted Nano-11 and commercial multivalent influenza vaccinates, indicating that the Nano-11-KAg-poly(I:C) vaccine-induced immune response did not translate into significant cross-protection. This suggests the need for further studies using multivalent SwIAVs-KAg in the Nano-11-KAg- poly(I:C) formulation in pigs. The Nano-11-peptides-poly(I:C) vaccine reduced the challenge virus load and lung lesions comparable to Nano-11-KAg-poly(I:C) vaccinates, consistent with our previous vaccine trial using PLGA encapsulated peptides vaccine [17].

## 5. Conclusions

In conclusion, the addition of poly(I:C) to a Nano-11-KAg vaccine formulation delivered IN augmented homologous, heterologous and heterosubtypic virus-specific humoral response in the airways and systemic, and Th1 and Th2 cytokines gene expression in the lung draining lymph nodes of pigs and provided partial cross-protective immunity. Future studies are aimed at improving the cross-protective efficacy of Nano-11-KAg vaccine by using multivalent SwIAVs KAg and split virus antigens along with other potent secondary adjuvants to better protect pigs against field virus infections compared to commercial multivalent SwIAV vaccines.

## 6. Ethics Statement

Accordance with the recommendations of Public Health Service Policy, United States Department of Agriculture Regulations, the National Research Council’s Guide for the Care and Use of Laboratory Animals, and the Federation of Animal Science Societies’ Guide for the Care and Use of Agricultural Animals in Agricultural Research and Teaching the animal study was carried out. We followed all relevant institutional, state, and federal regulations and policies regarding animal care and use at The Ohio State University. Pigs were maintained, samples collected, and euthanized in accordance with the approved protocol of the Institutional Animal Care and Use Committee at The Ohio State University (Protocol number 2015A00000120).

## Figures and Tables

**Table 1:** Sequences and isoelectric points of influenza virus-specific conserved T cell and B cell peptides.

S.No	Peptides	Sequence	Isoelectric Point
1	NP_44-52_ (T cell)	CTELKLSDY	4.37
2	PB_1542-551_ (T cell)	ATAQMALQLF	5.57
3	PB_1591-599_ (T cell)	VSDGGPNLY	3.8
4	M_136-45_ (T cell)	NTDLEALMEW	3.57
5	PB_2197-205_ (T cell)	VAGGTGSVY	5.49
6	HA_159-92_ (B cell)	SSDNGTCYPGDFIDYEELRE QLSSVSSFERFEIF	3.89
7	HA_187-120_ (B cell)	NSENGTCYPGDFIDYEELRE QLSSVSSFEKFEIF	3.94
8	HA_1101-134_ (B cell)	NPENGTCYPGYFADYEELR EQLSSVSSFERFEIF	4.06
9	M2e (B cell)	SLLTEVETPIRNGWECKCN DSSD	4.18
10	HA_276-130_ (B cell)	RIENLNKKVDDGFLDIWTY NAELLVLLENERTLDYHDS NVKNLYEKVRSQLKNNA	5.18

**Table 2:** Sequence of the primers used in qRT-PCR analyses.

S.No	Oligo Name	Sequence (5′ → 3′)
1	*β-actin*	CAGCCTCCTGAAACTGGAATAT (F) TCAGCAACAAGGTCTACAATCC (R)
2	*TNF-* *α*	CGTTGTAGCCAATGTCAAAGCC (F) TGCCCAGATTCAGCAAAGTCCA (R)
3	*IL-1ß*	TCTGTACCTGTCTTGTGTGATG (F) GCTTCTCCATGTCCCTCTTT (R)
4	*IL-13*	GTCATTGCTCTCACCTGCTT (F) TTGGTGTCTCGGATGTGCTT (R)
5	*IL-10*	GCATCCACTTCCAGGCCA (F) CTTCCTCATCTTCATCGTCA (R)
6	*GATA3*	TGCGGGCTCTACCACAAAAT (F) TAACCCGAGTAAAATGTGC (R)
7	*IL-2*	GATTTACAGTTGCTTTTGAA (F) GTTGAGTAGATGCTTTGACA (R)
8	*IL-6*	CCAGGAACCCAGCTATGAAC (F) CTGCACAGCCTCGACATT (R)
